# The Etiology, Diagnosis and Treatment of Diphtheria1Read before the Rochester Pathological Society, March 14, 1895.

**Published:** 1895-06

**Authors:** W. L. Conklin

**Affiliations:** 96 South Avenue; Rochester, N. Y.


### Buffalo Medical and Surgical Journal

VOL. XXXIV. JUNE, 1895. No. 11.

### Original Communications.

### THE ETIOLOGY, DIAGNOSIS AND TREATMENT OF DIPHTHERIA.[1]

BY W. L. CONKLIN, M. D., Rochester, N. Y.

THE subject of diphtheria is so familiar to all that it would seem a hopeless task to undertake to present any new thoughts in relation to it for your consideration. Much that is said must be a repetition of what has already been said; but at the present time, when we hear so frequently of methods of diagnosis and treatment which are of comparatively recent origin, it may be of interest to review the older ideas and clinical experiences, and by a comparison of results make an effort to determine how much advance has recently been made in the direction of greater accuracy in our knowledge of the origin of this dread malady, and increased facilities for its prompt diagnosis and successful treatment. While there is very much of interest pertaining to other phases of the subject, I shall confine myself, as far as possible, to the consideration of the etiology, diagnosis and treatment.

For over twenty-five years pathologists have believed in the germ origin of diphtheria, and from time to time have thought that they had discovered the specific microbe. In 1868, Oertel and others conducted experiments which convinced them that the micrococcus or globular bacterium was the cause of the disease. Five years later, Cohn named it "micrococcus diphtheriticus." About the same time, Klebs and Eberth regarded septic micrococci and the so-called diphtheritic micrococci as identical. The same opinion was expressed by Drs. Wood and Formad ten years later, after experiments made by them for the National Board of Health. In 1874, Billroth asserted that "the so-called pathogenic bacteria

1. Read before the Rochester Pathological Society, March 14, 1895.

of diseases are positively identical with those found in putrefying dead tissues." This assertion was also made by Drs. Curtis and Satterthwaite, after investigations and experiments made for the New York Board of Health. It was evidently necessary to look further for the specific germ of diphtheria.

In 1883, Klebs described a bacillus which he found present in nearly all fatal cases of diphtheria. In 1884, the same bacillus was described by Löffler, who inoculated guinea-pigs with a pure culture, producing death with the characteristic lesions of diphtheria. Further investigation led to the conclusion that the bacilli remain, for the most part at least, at the point of local manifestation of the disease, and that the toxic symptoms are due to the production by them of ptomaines or toxines which are absorbed by the lymphatics.

The Klebs-Löffler bacilli are described by Thompson and Park and Beebe, as "somewhat shorter than the tubercle bacilli, but much broader, and with thickened or clubbed extremities" as a rule. They are straight or slightly curved and non-motile. The bacilli possess no spores, but have in them highly refractile bodies. With an alkaline solution of methyl-blue they stain in a peculiar and characteristic way, as certain oval bodies, situated in the ends or in the central portion, stain much more intensely than the rest of the bacillus.

In 1888, the investigations and experiments of D'Espine, Roux and Yersin added much valuable proof to that already secured. The two latter found that filtered cultures of diphtheria bacilli injected beneath the skin of guinea-pigs caused their death ; showing the virulence of certain products resulting from the growth of the bacilli. They also observed true paralysis following the injection in animals when a fatal result was sufficiently delayed. They draw the following conclusion : "The occurrence of these paralyses, following the introduction of the bacilli of Klebs and Löffler, completes the resemblance of the experimental disease to the natural malady, and establishes with certainty the specific *rôle* of this bacillus." Welch says : "All the conditions have been fulfilled for diphtheria which are necessary for the most rigid proof of the dependence of an infective disease upon a given microörganism — namely, the constant presence of this organism in the lesions of the disease, the isolation of the organism in pure culture, the reproduction of the disease by inoculations of pure cultures, and similar distribution of the organism in the experimental and the natural disease." He adds : "In view of these facts we must agree with Prudden that we are now justified in saying that the name diphtheria, or, at least, primary diphtheria, should be applied, and exclusively applied, to that acute infectious disease, usually associated with a pseudo-membranous affection of the mucous membrane, which is primarily caused by the bacillus, called the bacillus diphtheriæ by Löffler."

If the Klebs-Löffler bacillus is essential to the existence of true diphtheria, so that the determination of its presence or absence in a doubtful case settles the question of diagnosis, then a great advance has been made in our knowledge of the disease, and one which cannot fail to be of the utmost practical importance. That we now have abundant evidence of such a relationship is very generally conceded, though still questioned by a few.

The importance of making a correct diagnosis, as early as possible, in throat troubles, cannot be too strongly emphasized. The more promptly the disease is recognized, the patient isolated, if necessary, and proper treatment begun, the greater the probability of recovery and smaller the danger of further infection.

If the physician is called to a well-marked case of pharyngeal diphtheria, after the pseudo-membrane has begun to form and toxic symptoms have appeared, there should be no difficulty in making a correct diagnosis at once. But, if called to this same case before there is anything characteristic in the appearance of the throat or alarming in the constitutional disturbance, he may find a condition present which will make him suspicious of serious trouble ; still, if he depends entirely upon the appearance of the throat, he may be unable to make a positive diagnosis until the characteristic membrane appears and there is evidence of grave general disturbance. Or, if the case be one in which the disease is primarily laryngeal or nasal, it is often very difficult to make an early diagnosis.

Some observers still believe that membranous croup is entirely distinct from laryngeal diphtheria, and so not contagious. If this be true, how important it is that such cases should be distinguished as promptly as possible from those which are true laryngeal diphtheria. Both are conditions of great danger to the patient, but, if the theory of their distinct character is correct, one calls for isolation and stringent measures to prevent the spread of the disease, while with the other no such precautions are necessary. No doubt most cases of follicular tonsilitis may readily be distinguished from diphtheria, even when the patches of secretion which occupy the crypts of the tonsil have coalesced. But who has not been uncertain as to diagnosis in such cases. That there are not a few cases of so-called pseudo-diphtheria, in which the macroscopic appearance of the membrane closely resembles that of true diphtheria, is now generally conceded. This condition is of frequent occurrence in cases of scarlet-fever and measles, and it is important to distinguish it from true diphtheria developing as a complication of those diseases.

Thus it appears that the early diagnosis of diphtheria is of great practical importance and that in the past it has been, in some instances, a very difficult matter. No one can read a report of one year's work in the New York bacteriological laboratory without being impressed with the evidence which it affords of the value of bacteriological examination of cases of diphtheria, not only as a means of diagnosis, but also for the purpose of determining when quarantine is no longer necessary. Such a report, written by Dr. William Park, bacteriological diagnostician and inspector of diphtheria, and Alfred L. Beebe, inspector of bacteriology, appeared in the *Medical Record*, September 29, 1894. It contains so much of interest relating to my subject that I shall quote from it freely.

From May 4, 1893, to May 4, 1894, a bacteriological examination was made of 5,611 cases of suspected diphtheria. The Klebs-Löffler bacilli were present in 3,255, absent in 1,540, and 816 were considered doubtful, owing to various deviations from regular methods in obtaining the cultures, although no diphtheria bacilli were present. It was concluded that about 60 per cent. of the cases were true diphtheria and 40 per cent. false or pseudo-diphtheria. In 1,625 cases of true diphtheria the age and mortality were ascertained. The number of cases increased with each year to the fourth. The mortality rate from the first to the fourth was 45 per cent.; from the fifth to the seventh 33 per cent.

Observations are recorded which have an important bearing upon the question of the identity or non-identity of membranous croup and laryngeal diphtheria. They are, in brief, as follows:

In 286 cases, 283 children and three adults, the disease was chiefly or entirely confined to the larynx or bronchi. In 229 of the 286 cases, 80 per cent., Klebs-Löffler bacilli were found. Seventy-three per cent. of these 229 cases showed no false membrane above the larynx. In fifty-seven cases no Klebs-Löffler bacilli were found. Of these, seventeen cultures were unsatisfactory. Excluding these, there were forty of pseudo-diphtheria. Summary: Eighty per cent. of true diphtheria; of the remaining 20 per cent., 14 per cent. only were certainly not diphtheria.

Up to the time these investigations were made, membranous croup had not been considered a contagious disease by the New York Board of Health, but the facts brought out resulted in a change in the sanitary code, June 6, 1894, by which the report of such cases was required. The same regulation was adopted by our own Board of Health January 19, 1894. In view of these facts it would seem a wise and conservative view of the subject to consider every case of true croup contagious, until the absence of the Löffler bacillus is proven by bacteriological examination.

The following statement is made regarding the reliability of such examinations for diagnostic purposes:

> In cases in which the disease is confined to the larynx or bronchi, and where, therefore, there is no visible exudate against which the swab can be rubbed, surprisingly accurate results can be obtained from the examination of cultures, but in a certain proportion of cases no diphtheria bacilli will be found in the first culture, and yet will be abundantly present in later ones, the bacilli having probably been coughed up more freely as the disease progressed. We believe, therefore, that absolute reliance for a diagnosis cannot be placed upon a negative result in a single culture from the pharynx in purely laryngeal cases.

The differential diagnosis between diphtheria and so-called pseudo-diphtheria is of great importance. The latter condition may be defined as an inflammation of the mucous membrane of the throat, accompanied by the formation of pseudo-membrane which resembles that of true diphtheria, in which the Klebs-Löffler bacilli are absent. Such cases are characterized by the presence of various forms of micrococci, especially streptococci, which are thought to be the cause of the disease, when associated with conditions favorable to the development of throat troubles. Various observers in Berlin, Paris, Switzerland, New York and Boston have found from 20 to 50 per cent. of pseudo-diphtheria among cases of suspected diphtheria. The difference of mortality rate in the two diseases is very striking. In hospital cases characterized by the presence of the Klebs-Löffler bacillus, the mortality rate has varied from 25 to 70 per cent. before the introduction of antitoxin, while in suspected cases in which this bacillus was absent it has varied from nothing to 20 per cent.

Among 450 cases of pseudo-diphtheria carefully investigated by the New York Board of Health, there were eleven deaths, a mortality of 2½ per cent. Forty-two of the 450 cases were associated with scarlet-fever. Of these, four died. Six were associated with measles and all of these recovered. One of the fatal cases was a man seventy years of age, who had a valvular lesion of the heart. Another adult died of septicemia. The larynx was affected in all of the five fatal uncomplicated cases in children under five years of age, and in three there was more or less broncho-pneumonia. One hundred and thirteen of these cases, occurring in 100 families, were carefully investigated in reference to the question of contagion. In only fourteen was the relationship with another case, so far as ascertained, such as to point to contagion as a probable cause. In nine of the 100 families more than one case occurred, but secondary cases seemed to develop as frequently when the patient was isolated as when no precautions were taken.

Careful investigation would seem to show that streptococci, in limited numbers, are present in many healthy throats, and that when they bear a causative relationship to the morbid condition called pseudo-diphtheria, their number is greatly increased and atmospheric conditions, a depressed state of the general health, and other disturbing elements are added factors.

It will be noticed that in the reports of work in the New York laboratory, all cases of suspected diphtheria are classified, after bacteriological examination, as either true diphtheria or pseudo-diphtheria. It would seem that this term, "pseudo-diphtheria," is objectionable, because somewhat misleading, and that if used at all, it should be applied only to those cases in which the bacillus is found which was first described by Hofmann, and afterward studied by Roux and Yersin and Escherich, and not to the large number of cases in which the streptococci or staphylococci predominate.

There is still a difference of opinion in regard to the so-called pseudo-diphtheria bacilli, and by some observers they are considered identical with Klebs-Löffler bacilli which have lost their virulence. Until more accurate and conclusive observations can be made, it would seem better to avoid the use of the term "pseudo-diphtheria" in the classification of suspected cases.

In the Paris hospitals, cases are classified, as suggested by Löffler, as "diphtheria pure, diphtheria associated with streptococci or staphylococci or both, and simple anginas." Patients under Prof. Koch's care are classified as pure diphtheria, diphtheria with toxemia, and diphtheria with mixed infection. For the purpose of determining whether a suspected case should be quarantined or not, classification as diphtheria or not diphtheria is sufficient. This is the plan followed by Prof. Dodge, but, in view of the fact that there seems to be abundant evidence to prove that cases in which there is a mixed infection of Klebs-Löffler bacilli, streptococci and staphylococci are, as a rule, more severe, it might be of help to the physician if, in the report to the health officer, the existence of such an infection was mentioned when present.

Considering the fact that so short a time has elapsed since bacteriological examination of suspected cases of diphtheria was first practised as a means of diagnosis, there is a large amount of convincing evidence as to its practical value.

A recent visit to the biological laboratory at the university convinced me that in the care and thoroughness with which bacteriological examinations are made, as in other departments of its work, the Rochester Board of Health and its officers are progressive and doing much to conserve the public health. The report of the board for February shows that of sixty-nine cases of suspected diphtheria, fifty-one, about 74 per cent., were proved diphtheria by bacteriological examination. For the purpose of determining the duration of quarantine, 251 cultures were made. The average duration of quarantine, as determined by bacteriological examination, was nineteen days ; the longest period forty-two days, the shortest period six days.

In discussing the treatment of diphtheria, the subject of prophylaxis merits careful consideration, for in no other disease are preventive measures more important, or more likely to be successful if thoroughly carried out. Great advance has been made in preventive medicine in recent years, and the public, as well as the medical profession, is fast learning to appreciate more fully, its importance ; but is there not some ground for the assertion that we have been slower to realize the urgent need of preventive measures in dealing with what are sometimes called the minor infectious diseases, including diphtheria and the acute exanthemata of childhood, than in contending with small-pox, cholera or yellow-fever ? Nearly every city has its small-pox hospital, but how few, comparatively, have a hospital for the treatment of diphtheria ; and, yet, how much greater the mortality from the latter than from the former disease. Dr. Kinyoun, of the Marine Hospital Service, in a paper entitled The Prevention of Diphtheria, says:

We have provided hospitals for the sick with nearly every other disease, and receive the patient without question, but when a case of diphtheria presents itself the doors are closed, and if, perchance, there still remains any charity for this class of sufferers, the accommodations are such as not to be designated as hospital or hospitable. Because one is unfortunate enough to contract a disease, does it follow that it is not the proper thing to have diphtheria or small-pox, while it is highly so to suffer from erysipelas or pneumonia, or from our time-honored associate and hightly-respected protégé, tuberculosis.

He adds:

I am glad to say that the question of providing facilities for the care and treatment of this class of maladies is interesting many. But here and there, over our broad land, we observe scenes transacted which are, in this enlightened age, barbaric and a blot on our civilization.

The need of such a hospital for Rochester has long been recognized by physicians, and the efforts of the health officer and others to awaken a general interest in the subject will receive the hearty endorsement of the profession.

The subject of prophylaxis will be considered under the following divisions: (1) Isolation of patient; (2) disinfection of patient, apartment and clothing; (3) precautions to be observed during convalescence; (4) immunization with diphtheria antitoxin.

While diphtheria is highly contagious, the area of direct contagion is said to be limited to a few feet. Isolation of the patient, then, in a room from which all unnecessary articles have been removed, and distant, if possible, from those occupied by other children in the family, will do much toward preventing the spread of the disease. If nourishment and whatever else may be required by patient or nurse can be left in an intervening vacant room, so that it shall not be necessary for the nurse to go directly from the sick-room to the living-rooms of the family, there will be still less danger of others being infected. Dr. J. Lewis Smith states that:

Dr. H. B. Baker has published statistics showing that in 102 outbreaks of diphtheria the average number of cases where disinfection and isolation, one or both, were neglected was sixteen, and the average deaths 3.26, while in 116 outbreaks in which isolation and disinfection were enforced, the average number of cases per outbreak was 2.86 and the average deaths 0.66. Therefore, these precautionary measures prevented thirteen cases and 2.57 deaths for each outbreak; in the total, 1,545 cases and 298 deaths.

As diphtheria is contracted, in the large majority of cases, by breathing air infected with Klebs-Löffler bacilli, the frequent and thorough disinfection of the throat will, by limiting to some extent the multiplication of these bacilli, do much to limit the spread of the disease. It has been wisely said that, "As far as practicable, we should deal with the infectious agents at their point of origin and under all circumstances act promptly."

As such disinfection, when secured by means suited to the age and condition of the patient, has a favorable effect upon the disease, it is of two-fold importance and will be considered further on under the head of treatment.

Various disinfectants may be used in the room, but it cannot be too strongly impressed upon the attendants that it is better and easier to replace infected air with that which is pure than it is to disinfect that which is contaminated. In a word, insist on as thorough and constant ventilation as it is possible to secure. The vapor of cresoline or of a mixture of carbolic acid, eucalyptus and turpentine may be of service, but should never be substituted for the more important preventive measure, thorough ventilation.

After the case has terminated, the disinfection of the sick-room can best be accomplished by prolonged airing and thorough scrubbing of floor and walls, followed by the application of a 1 to 1,000 corrosive sublimate solution. If sulphur is burned, there must be an abundance of moisture in the room or its effect will be *nil.* After an outbreak of diphtheria in the maternity ward of the New York Infant Asylum, the ward was vacated and forty pounds of sulphur, two pounds to 100 cubic feet of air, were burned without the presence of moisture. After several hours windows and doors were opened and dust raised which was allowed to settle on culture media. The experiment proved that large numbers of bacilli were present, some of which were identical with those which had been found on the diphtheritic membrane. All articles of clothing and bedding which can be boiled may be thoroughly disinfected by this process. Dr. John S. Billings says: "The experience of large public laundries, and especially of laundries connected with hospitals for infectious diseases, such as that in Glasgow, shows that all germs of infectious diseases are thus destroyed, and that clothing of small-pox, typhus and other patients may be mingled and go through the boiling-vats without risk to the subsequent wearers." Infected clothing should be placed in water or a bichloride solution as promptly as possible, and remain there until it can be boiled. It will not then be likely to liberate disease germs. The physician should wear a gown, linen duster or sheet while in the room and thoroughly wash his hands before leaving it. It has been well said that "Medical asepsis is as essential as surgical asepsis, and we are just as remiss in our duties as practitioners when we fail to attend carefully to the matter of disinfection, as in allowing erysipelas or suppuration to exist in our surgical cases when we can prevent them."

It has long been known that so-called ambulatory cases were frequently the means of spreading the contagion, but it is only recently that bacteriologists have made the important discovery that convalescent cases are often a source of contagion long after all traces of membrane have disappeared from the throat. It is true that the number of Klebs-Löffler bacilli rapidly grows smaller as convalescence advances, and that some of them lose their virulence; still, in many cases, enough virulent bacilli remain to make it unsafe to remove the quarantine for several days. Indeed, it is as a means of determining when this can safely be done that bacteriological examinations are of great value in every case. In connection with the investigations referred to, an effort was made to determine how long virulent bacilli remain in the throat after the disappearance of the membrane. Various observers have found Klebs-Löffler bacilli, that were proven fully virulent by the inoculation of guinea-pigs, at periods ranging from three to fifteen days. Morse found the average time ten days and Tobiesen nine days. In sixteen cases from which cultures were examined in the New York laboratory, virulent bacilli were found at periods ranging from six to fifty days, but of 605 consecutive cases no bacilli were found after three days from the disappearance of the membrane in 304, and in the remaining 301 the time varied from seven days to nine weeks. The wisdom of applying this test in every case, before quarantine is removed, cannot be doubted.

So frequently have virulent Klebs-Löffler bacilli been found in the healthy throats of those in constant attendance upon cases of diphtheria, it would be a wise preventive measure to determine by bacteriological examination whether the throats of nurse and members of the family are infected before permitting them to mingle freely with others. Escherich reports the following case, which illustrates the importance of such a precaution: "It was noticed among the children coming under the care of a certain apparently healthy nurse, a number of cases of diphtheria were developing. A bacteriological examination being made, her throat was found to contain very numerous virulent diphtheria bacilli."

In view of the fact that the length of time during which virulent bacilli remain in the throat is shorter, as a rule, in cases which are persistently treated with antiseptic applications, would it not be well to continue their use in all convalescent cases, until the bacilli have entirely disappeared ? Would it not be well, also, by way of prophylaxis, to direct those who are caring for the patient to spray the throat frequently with a weak bichloride solution, or some other disinfectant, in order that they may be less likely to contract the disease themselves, or harbor in their healthy throats bacilli which may prove a source of infection to others.

The assertion is made by careful observers that those who have been exposed to diphtheria may be rendered immune by one injection of antitoxin. If this is true, it is evidently a prophylactic measure of the greatest importance. Katz and others claim failure in only 10 per cent. of preventive inoculations, while Dr. Kinyoun says : "In every instance, whether in hospital or in homes, there has been no record of failure to protect." He adds : "The future possibilities in this direction cannot be overestimated, as we have in the serum the almost absolute preventive of epidemics of diphtheria."

We are told that twenty centuries ago Asclipiades scarified the tonsils and performed laryngotomy for the relief of respiration, and that "it is supposed he treated membranous croup and probably diphtheria." From that day to this, physicians have tried to solve the problem of the successful treatment of this dread disease, but it must be acknowledged that too often their best efforts have been of no avail. All honor to the American physician who has made it possible to lessen the fatality of the most dangerous form of the disease and to relieve, in so great a degree, the sufferings of many cases in which recovery is impossible. Still, with all that has been gained through the use of tracheotomy, intubation, germicides and the many valuable remedies of recent years, the mortality rate has remained very high, as the following statement, taken from *Pepper's Practice*, published less than two years ago, will show : "The death-rate of diphtheria varies with different epidemics. It sometimes exceeds 40 per cent. and has even reached 76 per cent. With 900 cases recently treated in Strasbourg the mortality was 46.7 per cent. . . . . . . Despite every effort for the control of diphtheria, the death-rate has remained undiminished for many years."

It may be safely asserted that if this last statement should be made today, it would not go unchallenged, for, while it is yet too early to make many positive assertions in regard to the new remedy which is attracting so much attention, it is not too much to say that since diphtheria antitoxin has been so generally used in the large European hospitals, the mortality rate reported by them is very much less than that of former years. It cannot be denied that there sometimes exists a tendency to grow over-enthusiastic in regard to new remedies which give even slight promise of aid in the treatment of this disease. The temptation is strong to believe that true which we would like to have true, without first demanding conclusive evidence in its favor. On the other hand, it is possible to be over-sceptical in regard to the utility of this, as well as of other remedies. Diphtheria antitoxin has now been on trial for several months, and all have read reports, favorable or otherwise, of the results obtained. I should like to group a few of these reports, gathered from various sources, and feel sure that such a grouping of evidence will show that the verdict is favorable, and that we have in antitoxin a remedy, the value of which has not, as a rule, been overestimated. At the Eastern Hospital, London, there were treated in 1893, 397 cases of diphtheria in children under fifteen years of age. Of these, 166, 41.8 per cent., died. From September, 14, 1894, to October 22, 1894, seventy-two cases were treated without serum, with a mortality of 38.8 per cent. From October 23, 1894, to November 27, 1894, seventy-two were treated with serum, with a mortality of 19.4 per cent. It is stated that the cases treated with serum were above rather than below the average severity.

In one of the Berlin hospitals the mortality rate of diphtheria from January 1 to March 14, 1894, was 41.8 per cent. From March 14, to June 20, 1894, 128 cases were treated with antitoxin, with a mortality of 13.2 per cent. Prof. Baginsky says: "We have never had such a low mortality with our mildest epidemics, and our best form of treatment." Dr. Goodall, of the Eastern Hospital, says: "In the course of a three years' residence in the midst of diphtheria I have tried myself, or seen my colleagues try, a considerable number of drugs in that disease. In my experience no other remedy has been so efficacious as antitoxin." The average mortality of diphtheria in one of the children's hospitals of Paris during four years was 51.71 per cent. From February 1 to July 24, 1894, 448 patients were treated with serum. There were 109 deaths, a mortality of 24.5 per cent. During the same period 520 cases were treated without serum in the Trousseau Hospital with 316 deaths, a mortality of 60 per cent.

Dr. Fischer, of the Post-Graduate, New York, reports thirty-four cases treated with antitoxin. Thirty of these he designates malignant and four mild. Two died, a mortality of 5.8 per cent. The diagnosis in most of these cases was verified by bacteriological examination. At the International Congress at Buda-Pest, last September, Prof. Roux reported 120 cases with nine deaths, a mortality of 7½ per cent. Virchow has said much in criticism of the new remedy, but quite recently he has become convinced of its value and has given it his full endorsement. At a meeting of the Berlin Medical Society he gave the results obtained by the serum treatment in the Emperor and Empress Hospital, and remarked that all theoretical considerations must give way to the force of these figures.

Dr. J. J. Kinyoun, of the Marine Hospital service, has recently visited Paris and Berlin to make an official investigation of the serum-therapy of diphtheria. He was afforded every facility for the study of the subject by Prof. Roux, of the Pasteur Institute, Prof. Baginsky, of the Children's Hospital, Berlin, and Prof. Koch, of the Institute for Infectious Diseases, and in his excellent report to the Supervising Surgeon-General, M. H. S., he says: "From my observations I can but corroborate the statements already published. I have been able to follow the cases from the time they entered the hospital until their discharge, noting everything which has been done. I have tried hard to find fault, to pick flaws in the statistics, but have signally failed. The work must stand for itself."

The suggestion has been made by Mr. Lennox Brown and others that the use of antitoxin has a tendency to produce renal complications. Indeed, this is about the only serious objection which has been made to its use. If this be a valid objection, it is hard to reconcile it with the following facts: A large number of cases of suspected diphtheria were treated with antitoxin in the Eastern Hospital, London. In not one of the non-diphtheritic cases among these was there any albuminuria. Dr. Kinyoun quotes

Roux as saying, that "before treatment with injections of antitoxin, albumen was found to be present in two-thirds of the cases of pure diphtheritic angina. After treatment it was found in scarcely half the cases.

In presenting the following outline of treatment for your consideration, I wish to emphasize the statement that it is only an outline, to be added to and modified according to the necessities and requirements of individual cases. In a suspected case, insist on isolation of the patient at once, making such explanations as will quiet the fears of friends. Make a culture from the throat, and secure a bacteriological examination if possible, and as soon as possible. Make a second culture if the examination of the first gives a negative result, where there are indications of primary laryngeal diphtheria. If the suspicion of diphtheria is verified by the bacteriological examination, inject, with strict antiseptic precautions, from 15 to 20 c. c. of serum. The repetition of this injection on the two or three following days should depend upon the condition of the patient. If it is not evident that the disease is already yielding, other injections are indicated and should be used as required, until improvement or failure is evident. Some of the indications for a repetition are, coincident rise of pulse-rate and temperature; increase of toxic symptoms; involvement of larynx and bronchi and appearance or increase of albumen. There seems to be abundant evidence that if the bacteriological examination shows an association of streptococci, in large numbers, with the Klebs-Löffler bacilli, either in pharyngeal diphtheria or membranous croup, the attack will be likely to be more severe. Dr. Kinyoun says of the Paris hospitals: "Great stress is laid upon the class of cases in which the diphtheria is complicated with the pus cocci, especially so when the streptococci are present. The prognosis in these is, from the very commencement, looked upon as grave." Such an association would be another indication for repeated injections of antitoxin. Variations in temperature have not usually been considered of much significance in the treatment of diphtheria, but the importance of such variations in connection with the serum treatment is generally conceded. Dr. Kinyoun has added to the article referred to, a number of temperature charts, which are of interest as an indication of the temperature variations during the period of active treatment and until the termina tion of the case. The charts include cases of pharyngeal diphtheria, membranous croup, diphtheria associated with streptococci, and simple angina. An initial rise will be noticed, in many cases, on the day following the first injection.

The indications for intubation are the same with this form of treatment as with any other, and should be as promptly met; but attention has been called to the fact, that in the hospitals of Paris and Berlin laryngeal stenosis is not so frequently met with as it was before antitoxin was used. If the serum treatment is used, it does not seem necessary or, perhaps, advisable to follow closely any plan of internal medication; but there can be no objection to the use of such drugs as may be called for by the special requirements of the case. The same may be said regarding the use of stimulants. The use of antitoxin does not preclude the employment of many local disinfectants, though Roux objects to the use of solutions of bichloride and carbolic acid. He advises irrigating the throat three times a day with a solution of boracic acid or of Labarraques solution, 1½ ozs. to a quart of water. Löffler recommends a solution composed of menthol, 10 grains; toluine, 36 c. c.; then add creolin, 2 c. c.; iron chloride solution (4 per cent.), 4 c. c.; alcohol q. s., 100 c. c. This is applied every three or four hours by means of a pledget of cotton, after the mucus has been wiped from the throat. This solution is put up by Parke, Davis & Co. A similar solution was used in ninety-six cases, three-fourths of which had been verified by bacteriological examination, without a single death. Peroxide of hydrogen, in varying strengths, has been used by many with success, and is doubtless one of the most valuable of the local applications. When a thorough irrigation of nose and throat can be accomplished, it is of great value. By the use of a rubber sheet this can sometimes be done while the patient is in a recumbent position. Great care must be exercised in the use of local applications to adapt the remedy and method of using it to the individual case.

The successful management of a case of diphtheria depends, in a very large degree, upon the success with which nourishment is given and its assimilation secured. It will be readily seen, however, that the urgent need of nourishing the patient, to the greatest possible extent, grows out of the fact that the rapid absorption of ptomaines or toxines produces a condition of profound depression of the vital powers. If, by the prompt use of antitoxin and the local application of germicides, this toxic condition can be prevented to a large degree, there may not be the same necessity for what is sometimes a forced alimentation, as has been evident in connection with other methods of treatment. Still, it is certainly wise to give as much nourishment as can be properly assimilated in the treatment of every case of diphtheria.

It would seem that within the last few months great advance has been made, both in the possession of means for accurate and early diagnosis, and in the discovery of a remedy which has already accomplished much and gives reasonable promise of still greater things in the future. It is true that even though the utmost care is exercised, errors in diagnosis will occasionally be made, and equally true that even though the remedy be applied at the earliest possible moment, some cases will prove fatal. But, when the evidence is carefully considered, is it not enough to make us hopeful, at least, that in the future diphtheria will not prove to be the dreadful scourge which it has been in the past?

96 South Avenue.

